# Meningeal and cortical grey matter pathology in multiple sclerosis

**DOI:** 10.1186/1471-2377-12-11

**Published:** 2012-03-07

**Authors:** Bogdan F Gh Popescu, Claudia F Lucchinetti

**Affiliations:** 1 Department of Anatomy and Cell Biology, University of Saskatchewan, 107 Wiggins Road, Saskatoon, SK S7N 5E5, Canada; 2 Cameco MS Neuroscience Research Center, Saskatoon City Hospital, 701 Queen Street, Saskatoon, SK S7K 0M7, Canada; 3 Department of Neurology, Mayo Clinic, 200 First Street SW, Rochester, MN 55905, USA

## Abstract

Although historically considered a disease primarily affecting the white matter of the central nervous system, recent pathological and imaging studies have established that cortical demyelination is common in multiple sclerosis and more extensive than previously appreciated. Subpial, intracortical and leukocortical lesions are the three cortical lesion types described in the cerebral and cerebellar cortices of patients with multiple sclerosis. Cortical demyelination may be the pathological substrate of progression, and an important pathologic correlate of irreversible disability, epilepsy and cognitive impairment. Cortical lesions of chronic progressive multiple sclerosis patients are characterized by a dominant effector cell population of microglia, by the absence of macrophagic and leukocytic inflammatory infiltrates, and may be driven in part by organized meningeal inflammatory infiltrates. Cortical demyelination is also present and common in early MS, is topographically associated with prominent meningeal inflammation and may even precede the appearance of classic white matter plaques in some MS patients. However, the pathology of early cortical lesions is different than that of chronic MS in the sense that early cortical lesions are highly inflammatory, suggesting that neurodegeneration in MS occurs on an inflammatory background and raising interesting questions regarding the role of cortical demyelination and meningeal inflammation in initiating and perpetuating the disease process in early MS.

## Review

Multiple sclerosis (MS), a chronic idiopathic inflammatory demyelinating disease, is the most common cause of nontraumatic disability in young adults [1]. Focal areas of demyelination and relative axonal preservation on a background of inflammation and gliosis represent the pathological features of MS [2,3].

Historically, MS has been considered a disease primarily affecting the CNS white matter and its pathological features were beautifully described and illustrated by Professor Jean-Martin Charcot, an illustrious French neurologist and pathologist, in his famous "Lectures on the Diseases of the Nervous System" delivered at the Salpêtrière in 1868 [2]. In lecture VI, entitled "Disseminated Sclerosis. Pathological Anatomy", besides acknowledging the involvement of the cerebral and cerebellar white matter, deep grey matter, brainstem, and spinal cord white and grey matter, Professor Charcot also pointed to the existence of the cortical demyelinated lesions: "...*the patches are very rarely found on the grey substance of the convolutions*" [2]. Because of their poor visualization when using conventional histological methods [4], the study of cortical lesions has largely been disregarded although early post-Charcot neuropathological studies have increasingly recognized the involvement of grey matter in MS [5,6].

While white matter lesion load has been able to explain only part of the clinical deficits seen in MS patients, cognitive impairment and seizures could be better explained by pathological processes affecting the grey matter [7-14]. It has also been shown that immunohistochemical techniques are superior to classical myelin histochemical staining methods (luxol fast blue) for detecting cortical demyelinating lesions [4]. Moreover, even among immunohistochemical markers for various myelin proteins, the use of antibodies specific to major myelin proteins is superior to antibodies against minor myelin proteins at detecting cortical demyelination. Therefore, armed with a clinical rationale and with the means to pathologically detect cortical lesions, the last decade has witnessed a surge in the interest allocated to cortical demyelination in MS and represented a "golden age" not only for neuropathological, but also for imaging and clinical studies on MS cortical pathology. Recent imaging and pathological studies have indeed confirmed that cortical demyelination is common in MS and more extensive than previously appreciated [4,15-21].

## Classification

The first pathological classification of cortical lesions has been proposed by Kidd *et al. *[17]. Seven cortical demyelinated lesion types have been described based on their location relative to the venous supply of the cerebral cortex. Type 1 lesions affect both the deep grey matter layers and the subadjacent white matter. Type 2 lesions involve all cortical layers without affecting the underlying white matter. Type 3 lesions involve only the upper cortical layers. Type 4 lesions only involve the subcortical U fibers. Type 5 lesions affect all grey matter layers as well as the subcortical white matter. Type 6 lesions are small demyelinated lesion that can occur in any part of the cortex. Type 7 lesions extend on both banks of a gyrus with or without involvement of the subcortical white matter. The majority of lesions (types 1, 4 and 5) arise within the territory of the principal vein [17], whose course begins in the white matter and passes through the cortex [22]. The remaining lesions are located in the territory drained by its branches or in the territory of the superficial veins [17].

**Figure 1:**
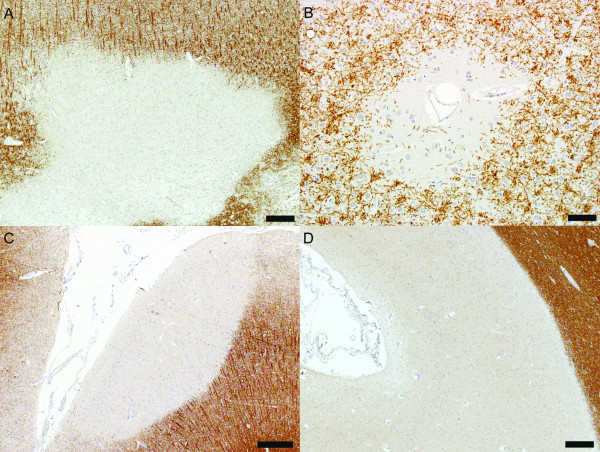
**Cortical demyelinated lesion types**. (A) Leukocortical lesion (PLP, scale bar = 100 μm); (B) Intracortical lesion (PLP, scale bar = 500 μm); (C) Subpial lesion affecting the superficial cortical layers (PLP, scale bar = 250 μm); (D) Subpial lesion involving all cortical layers without affecting the white matter (PLP, scale bar = 500 μm).

A simpler classification has been proposed by Peterson *et al. *and is based on the location of the cortical demyelinated lesions within the layers of the cortical grey matter [4,16]. Studying autopsy material from patients with chronic MS, the authors described four cortical lesion types [4,16]. Type I lesions, also called leukocortical lesions, involve the deeper layers of the grey matter as well as the subadjacent white matter at the grey/white matter junction with sparing of the superficial layers of the cortex (Figure 1A). Type II lesions, or intracortical lesions, are small demyelinated lesions centered on blood vessels and confined within the cortex, with sparing of both the superficial cortex and the white matter (Figure 1B). Type III lesions extend from the pial surface into the cortex, most often reaching the cortical layers 3 or 4 (Figure 1C). Type IV lesions extend to the entire width of the cortex without entering the subcortical white matter (Figure 1D) [4,16]. Type III and IV lesions are referred to as subpial lesions and can involve multiple adjacent gyri (general subpial demyelination) [4,16]. Subpial, intracortical and leukocortical demyelinating lesions have also been described in the cerebellar cortex and hippocampus [23-26].

While the latter classification is widely accepted currently, there are cortical demyelinated lesions that are unaccounted for by the present classification, highlighting the still incomplete understanding of their origin and evolution. We consider lesions affecting all 6 cortical layers with only marginal involvement of the white matter as subpial, rather than leukocortical demyelinated lesions [27]. Their subpial nature is supported by the centrifugal extension of the lesion from the pial surface into the cortex; the involvement of all cortical layers; the vast area of demyelination within the cortex with only limited involvement of the subcortical white matter; and the fact that the center of the lesion is in the grey and not in the white matter [27] as most often reported for leukocortical lesions [4,16]. How to classify a cortical lesion involving the whole grey matter width as well as a considerable region of the subadjacent white matter becomes even more complicated, and, theoretically, any of the following possibilities is feasible: a subpial lesion that extends into the white matter; a white matter lesions that extends into the cortex eventually reaching the pia; the coalescence of a subpial lesion with a white matter lesion; or the coalescence of a subpial lesion with a leukocortical lesion. While sometimes there are pathological cues that help in inferring the evolution of such a lesion [28], it is clear in most cases that its dynamics cannot be deduced from only one snapshot in the lesion's development that pathology offers.

## The Pathology of Cortical Demyelination and Meningeal Inflammation in Chronic Progressive MS

**Figure 2:**
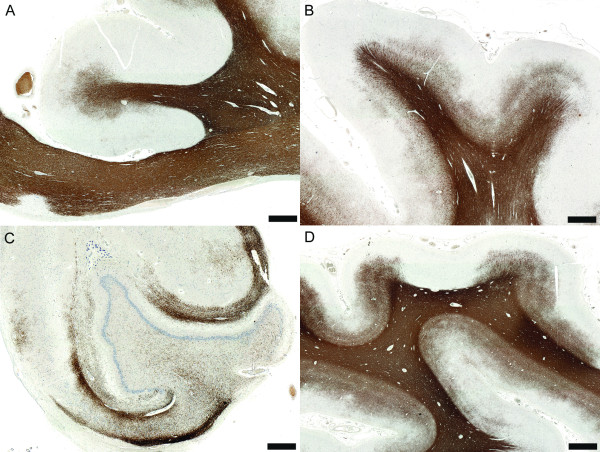
**Non-inflammatory cortical demyelination is prominent in chronic MS**. (A) Cortical demyelination in the cingulate gyrus (PLP, scale bar = 5 mm); (B) Cortical demyelination in the frontal lobe (PLP, scale bar = 5 mm); (C) Cortical demyelination in the hippocampus (PLP, scale bar = 2.5 mm); (D) Although the occipital lobe is less frequently affected, cortical demyelination can be very extensive in the occipital lobe of some MS patients (PLP, scale bar = 5 mm).

The most extensive cortical demyelination has been detected in the cingulate gyrus (Figure 2A), frontal (Figure 2B), temporal, insular and cerebellar cortices, as well as in the hippocampi (Figure 2C) of patients with progressive MS, while the paracentral lobule and the occipital lobe are less frequently affected but not spared (Figure 2D) [16,18,23-26,29]. Subpial demyelination preferentially involves the sulci suggesting that it may be induced by stagnant cerebrospinal fluid mediators [4]. Pathological studies have shown that cortical demyelination is prominent and extensive in primary progressive and secondary progressive MS, and in MS patients with cognitive deficits, suggesting it may be the pathological substrate of progression, and an important pathologic correlate of irreversible disability and cognitive impairment [16,18,25].

Similarly to white matter lesions, cortical demyelinated lesions studied in autopsy material from chronic MS patients are well-demarcated, and show oligodendrocyte, axonal and synaptic loss [4,15,16,20]. However, other studies described normal synaptic densities in cortical lesions [25,30].

In several other aspects, the pathology of cortical lesions has been reported to be fundamentally different, *i.e*. cortical demyelinated lesions are less inflammatory than white matter lesions. Purely cortical lesions reportedly lack the lymphocytic and macrophagic inflammatory infiltrates, complement deposition and blood-brain barrier breakdown [4,15,16]. Leukocortical lesions are more inflammatory than subpial and intracortical lesions, but less so than lesions in the white matter [4,15,16,25,30]. The majority of phagocytic cells in cortical lesions have the morphology of activated ramified microglia that appear in close apposition to neurites and neuronal cell bodies [4,15,16,25,30]. Neurons are also damaged as evidenced by neuronal atrophy and apoptosis, and a mild to moderate reduction of neuronal densities in the demyelinated MS cortex [4,16,20,30].

Meningeal inflammatory infiltrates have been extensively characterized in late-stage progressive MS. They are topographically associated with subpial lesions, resemble lymphoid follicular structures, predominate among patients with secondary progressive MS [31,32] and are immunoreactive for Epstein-Barr virus [33]. However, the identification of Epstein-Barr virus infection of meningeal B-cells and its potential role in the pathogenesis of MS remains controversial as these findings have not been confirmed by other studies [34,35]. Furthermore, ectopic B-cell follicle-like structures, topographically associated with subpial lesions, are located in the deep sulci of the temporal, cingulate, insula and frontal cortices of secondary progressive MS patients with accelerated clinical course [36]. Therefore, meningeal inflammatory aggregates are believed to contribute to both cortical demyelination and MS disease progression.

Thus, according to studies that have analyzed cortical pathology in autopsy material from chronic MS patients, cortical demyelination is devoid of inflammatory lymphocytes and macrophages and is driven in part by organized meningeal inflammatory infiltrates [4,15,16,31,36]. Corroborated with evidence for neuronal degeneration and glial loss [4,16,20,30], this has led to the suggestion that neurodegeneration in MS proceeds independent of inflammation. However, we have to stress again upon the fact that these studies have relied on post-mortem tissue analysis from MS patients with long-standing, end-stage disease, and that virtually nothing was known about the pathology of cortical demyelination in early MS.

## The Pathology of Cortical Demyelination and Meningeal Inflammation in Early MS

MS may involve the cortex either as classically demyelinated plaques or as neuronal loss and atrophy following retrograde degeneration from white matter lesions [6]. Although cortical lesions could occur secondary to white matter damage [37,38], recent histopathological studies have shown that cortical demyelination may occur spatially removed from and without obvious anatomical relationships to the white matter pathology [39]. Therefore, it is plausible that the cortex could represent an important early and/or initial target of the MS disease process.

MRI has confirmed that cortical lesions and atrophy are already present in early disease [19,40,41] and become more prominent during progressive MS [7,42]. Thirty seven percent of patients with clinically isolated syndrome show the presence of cortical lesions [43]. Cortical lesions are more numerous and cortical atrophy is more pronounced in patients with relapsing remitting MS and cognitive deficits than patients with relapsing remitting MS who do not have cognitive deficits [44]. Relapsing remitting MS patients with epilepsy also have more severe and rapidly evolving cortical lesion load and atrophy compared with relapsing remitting MS patients without epilepsy [45]. Also, a longitudinal MRS study of cortex in relapsing remitting MS has showed periodic peaks consistent with myelin breakdown [46], and increased apparent diffusion coefficients in normal appearing cortex, probably reflecting focal and diffuse cortical damage [40]. Thus, cortical demyelination is already present and common in early MS, and may represent the pathological substrate of cognitive impairment and epilepsy in relapsing remitting MS. Furthermore, MRI evidence of MS cortical onset [41] has been published supporting the hypothesis that cortical demyelination could even represent the earliest pathological event in some MS patients.

**Figure 3:**
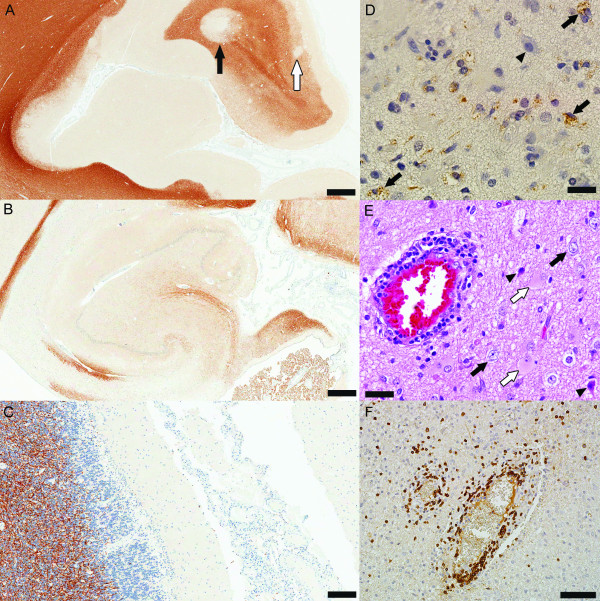
**Inflammatory cortical demyelination is present and common in early MS**. (A-C) Tissue obtained at autopsy form patients with acute MS: (A) Prominent cortical demyelination in the neocortex with the presence of all cortical lesion types: extensive subpial demyelination, intracortical demyelination (white arrow) and leukocortical demyelination (black arrow) (PLP, scale bar = 2.5 mm); (B) Cortical demyelination in the hippocampus (PLP, scale bar = 2.5 mm); (C) Subpial cerebellar demyelinated lesion (PLP, scale bar = 500 μm). (D-E) Inflammatory cortical demyelination in the cortex from biopsied patients: (D) Demyelinated cortex (arrowhead indicates a neuron) infiltrated by myelin-laden macrophages consistent with active demyelination (arrows) (PLP; scale bar = 50 μm); (E) Perivascular inflammation, degenerating neurons (arrowheads) scattered among normal-looking neurons (black arrows) and reactive astrocytes (white arrows) are present in cortical lesions in early MS (HE, scale bar = 50 μm); (F) Lymphocytic perivascular and diffuse inflammatory infiltrates in the demyelinated cortex (CD3, scale bar = 250 μm).

Recent research using brain biopsies obtained early in MS has indeed revealed that cortical demyelination and blood-brain barrier damage also occur early in disease (Figure 3A-C), even preceding the appearance of classic white matter plaques in some MS patients [21,27]. The majority of all cortical lesion types in early MS are highly inflammatory with intense myelin-laden macrophages (Figure 3D) and lymphocytic (Figure 3E-F) infiltrates similarly to active white matter lesions [21,27]. Lymphocytes, macrophages and microglia are seen in close apposition to neurons and neurites, suggesting a direct role of the inflammatory cells in neuronal damage (Figure 3E) [21,27]. All these differences can be explained by different lesional stages and suggest that the non-inflammatory character of chronic cortical demyelination may relate to long intervals between lesion formation and autopsy [47]. Similarly to chronic plaques, cortical lesions in early MS also show loss of oligodendrocyte, and axonal and neuronal injury (Figure 3E) most likely the result of the acute inflammatory insult [21,27]. The presence of inflammatory cortical demyelination in early MS argues against a primary neurodegenerative process at this stage of disease. A recently developed rodent EAE model for cortical demyelination demonstrated that cortical inflammation is an early, transient and rapidly resolving phenomenon [48]. Therefore, the rapid resolution of cortical inflammation may in part explain these apparent discordant findings with respect to the presence of inflammation in early versus chronic cortical demyelinating lesions. The fact that cortical demyelination is more extensive in progressive than early MS could be explained by the extremely fast and efficient remyelination of cortical lesions in early disease stages [49].

**Figure 4:**
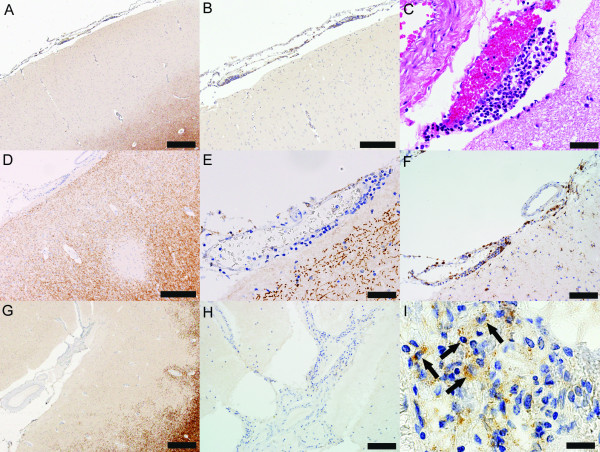
**Meningeal inflammation is topographically associated with cortical demyelination in early MS (autopsy tissue from patients with acute MS)**. (A) Subpial lesion overlaid by a perivascular meningeal inflammatory infiltrate (PLP, scale bar = 500 μm); (B) Higher magnification of (A) (PLP, scale bar = 250 μm); (C) Higher magnification of the perivascular meningeal infiltrate (HE, scale bar = 50 μm); (D) Intracortical lesion overlaid by perivascular meningeal inflammation (PLP, scale bar = 250 μm); (E) Higher magnification of the perivascular meningeal infiltrate (PLP, scale bar = 50 μm); (F) Macrophages are components of the perivascular meningeal infiltrate (KiM1P, scale bar = 100 μm); (G) Large subpial lesion underlying meningeal inflammation (PLP, scale bar = 500 μm); (H) Higher magnification of the meningeal inflammation (PLP, scale bar = 100 μm); (I) Myelin-laden macrophages are found in the meninges of patients with acute MS (PLP, scale bar = 20 μm).

Meningeal inflammation is prominent not only in chronic MS, but also in early MS. Both focal perivascular meningeal inflammation and diffuse meningeal inflammation are topographically, significantly and strongly associated with cortical lesions in early MS (Figure 4) [21]. By analogy with observations made in experimental autoimmune encephalomyelitis [50-52], it is plausible that early meningeal inflammation in MS, accompanied by inflammatory cytokine production in the subarachnoid space, could not only drive cortical demyelination, but also set the stage for subsequent subcortical white matter inflammation and demyelination [21].

## Conclusion

Although very prominent in chronic progressive MS [4,15,16,18], pathological and MRI evidence show that cortical demyelinated lesions are also present and common in early MS, and may be driven by meningeal inflammation [18,21,27,41,43-45]. Their inflammatory character suggests that neurodegeneration in MS runs on a background of inflammation [21,27]. Moreover, inflammatory cortical lesions may precede the appearance of classic white matter plaques at MS onset emphasizing the importance of considering demyelinating disease in the differential diagnosis of patients presenting with a solitary cortical enhancing lesion on MRI [27,41].

Although pathology has played a major role in understanding the pathophysiology of cortical demyelination, it is clear that the evolution of cortical lesions cannot be entirely understood from only one snapshot in the lesion's development that pathology offers. Conventional MRI techniques are not sensitive to detect intracortical and subpial lesions, but more recent imaging protocols using double inversion recovery and high field MRI have substantially improved their in vivo detection [53-57], and, without a doubt, will play, alongside pathology, a major role in fully elucidating the contribution of cortical demyelination to MS pathogenesis.

## Competing interests

Dr. Popescu declares that he has no competing interests.

Dr. Lucchinetti is listed as author and receives royalties for patent re: Aquaporin-4 associated antibodies for diagnosis of neuromyelitis optica; receives royalties from the publication of Blue Books of Neurology: Multiple Sclerosis 3 (Saunders Elsevier, 2010).

## Authors' contributions

Both authors were involved in designing, writing, and editing the manuscript, and reviewed and fully approved the final version of the manuscript for submission.

## Pre-publication history

The pre-publication history for this paper can be accessed here:

http://www.biomedcentral.com/1471-2377/12/11/prepub
